# Niche squeeze induced by climate change of the cold-tolerant subtropical montane *Podocarpus parlatorei*

**DOI:** 10.1098/rsos.180513

**Published:** 2018-11-28

**Authors:** María Paula Quiroga, Andrea C. Premoli, Thomas Kitzberger

**Affiliations:** INIBIOMA, Universidad Nacional del Comahue, CONICET, Laboratorio Ecotono, Quintral 1250, Bariloche 8400, Argentina

**Keywords:** cold-tolerant species, future environmental niche models, montane subtropical habitats, Yungas

## Abstract

Under changing climates, the persistence of montane subtropical taxa may be threatened as suitable habitats decrease with elevation. We developed future environmental niche models (ENNMs) for *Podocarpus parlatorei,* the only conifer from southern Yungas in South America, and projected it onto two greenhouse gas concentration scenarios based on 13 global climate models for the years 2050 and 2070. Modelling identified that *P. parlatorei* is sensitive and restricted to a relatively narrow range of both warm season temperature and precipitation. By the mid-late twenty-first century areas of high suitability for *P. parlatorei* will not migrate but overall suitability will become substantially reduced across its whole range and surrounding areas. Despite extensive areas in high mountain ranges where the species may encounter thermally optimal conditions to potentially allow upward local migration, these same areas will likely become strongly aridified under future conditions. On the other hand, in lowland locations where rainfall levels will not change substantially (e.g. northern range), excessive warming will likely generate abiotic and biotic restrictions (e.g. competition with lowland species) for this cold-tolerant species. Urgent measures should be developed for the local long-term preservation of the gene pool of the unique conifer that characterizes Yungas forests for reasons of biodiversity conservation and ecosystem services.

## 1. Introduction

Tropical forests have shown a striking variability in their vulnerability to warming by climatic change [1]. Nonetheless, most such evidence comes from lowland tropical forests. Pessimistic models used realized thermal niches to predict strong impacts of warmer conditions without considering that upper thermal limits of the fundamental niches are mostly unknown. By contrast, more optimistic views suggest that evergreen lowland tropical forests are among the least vulnerable biomes to warming, given low absolute increases in temperature compared to upper thermal tolerance limits [2]. While high-elevation species may benefit from steep climate gradients in mountain regions, their persistence may be threatened by limited suitable habitat as available land area decreases with elevation [3]. Also, most predictions under climate change suggest a temperature increase and the displacement of the ‘equatorial limit’ of plant species towards higher, i.e. colder, latitudes [4–7], i.e. to the north and south in the Northern and Southern Hemispheres, respectively.

The effects of rapidly changing global climatic conditions on tropical montane forests have been the subject of much speculation [8–13]. Their consequences remain highly uncertain due to severe threats from direct human exploitation (e.g. logging, agricultural expansion) and the indirect influence of human-accelerated environmental change. An improved understanding of the factors that affect species' distributions of tropical assemblages is necessary in order to contribute to the management and protection of these forests [14].

Traits that enable species to persist in a particular ecological environment are often maintained over time, a phenomenon known as niche conservatism [15]. Unless they are capable of rapidly adapting or adjusting through plasticity and/or shifting their distributions poleward and/or upward as is required to remain within their thermal niches, many species are predicted to experience range contractions and high local population extinction risks [13,16–23]. It was also forecasted that as thermal zones are lifted to highland areas species’ ranges will shift upwards. Thus, populations formerly confined to cool mountaintops will tend to lose habitat typically consisting of highly unsuitable rocky substrates or will be virtually eradicated from the top of lower mountains as found in the Guayana Highlands [24]. On the other hand, global warming simulations show not only increased temperatures but also substantial drying trends in tropical regions of Central America, the Caribbean, and equatorial South America [25] which may significantly impact on species' ranges.

Potential range shifts of species tracking their optimum climate as well as the detection of populations at risk of local extinction in the short term can be successfully predicted using models such as ecological niche modelling [26] or environmental niche models (ENNMs). ENNMs are useful tools for projecting potential shifts in the distributions of suitable conditions for species [27], under the assumption that the species’ ecological niche does not evolve to meet changing scenarios [28] and that species' ranges are determined by climate [29].

Heterogeneous conditions along species’ ranges may result in locally adapted and thus genetically differentiated populations. Also among-population genetic divergence might be a consequence not only of differential selection pressures but also population isolation [30]. Thus, under changing scenarios, modification of distribution ranges may significantly impact on species' gene pool and population long-term persistence. This may include the loss of unique genetic variants and/or genetic diversity due to bottlenecks or founder effects, as well as modification of gene flow rates or potential for adaptation, which should be taken into consideration in management and conservation plans.

The Podocarpacae in South America is the exclusive gymnosperm group that inhabits subtropical and tropical montane and lowland forests. The 29 neotropical *Podocarpus* species account for nearly a third of the species currently recognized in the genus [31]. The podocarps are slow-growing tropical trees and are mostly restricted to montane forests [32]. In South America, tropical and subtropical cloud forests, known as Yungas, are distributed along the western slopes of the Andes where *Podocarpus parlatorei* Pilger. is the only conifer inhabiting the montane forests of northwestern Argentina and southern Bolivia.

The aim of this work is to estimate the short-term changes in the potential distribution of *P. parlatorei* for the mid-late twenty-first century and assess local population vulnerabilities under plausible climatic scenarios of greenhouse gas emissions. Here we modelled two future (year 2050 and 2070) potential range of *P. parlatorei* under contrasting emission scenarios (Representative Concentration Pathways, i.e. RCP2.6 and RCP8.5) based on 13 different available high-resolution Global Climate Models (GCMs). We discuss the results and potential long-term viability of the entire species and sampled populations along the range of *P. parlatorei* using previously published genetic data in order to guide conservation actions of such ecologically relevant montane species.

## 2. Material and methods

### 2.1. Studied species

*Podocarpus parlatorei* occurs in montane environments from 17 to 28° S latitude and elevations that vary from 1200 m.a.s.l. at its southern limit in Argentina to 3000 m.a.s.l. at its northern limit in Bolivia. It is considered a pioneer species, and abundant regeneration is associated with large-scale disturbances of anthropogenic and natural origin [33,34]. At its southern limit, *P. parlatorei* generally occurs as pure forests, whereas in the north it grows underneath the canopy of *Alnus acuminata*, *Cedrela angustifolia* and *Juglans australis* [35]. It is wind-pollinated, and fruits are dispersed by gravity and/or zoochory ([36]; R. Salinas, Environmental Secretary, Catamarca, Argentina, personal communication). The populations of *P. parlatorei* are naturally disjunct and populations are ecologically subdivided into northern, central and southern sectors that are genetically divergent from one another as a result of historical isolation [37,38]. In Bolivia, it grows on the Peruano–Boliviano Yungas and continues to the south in Argentina, on the Boliviano–Tucumano formation [39]. Ecologically, *P. parlatorei* is a montane and relatively cold-tolerant taxon and thus it can be considered relatively sensitive to warming both due to adverse abiotic effects and poor competition with lowland trees [40]. Previous studies based on environmental niche models suggest that during cooling trends during the last glacial maximum *P. parlatorei* expanded its range towards eastern and southern lowlands compared with its present-day high-elevation distribution [41]. Its conservation status is near threatened [42] and is listed as CITES Appendix 1, which includes species with maximum risk of extinction (https://cites.org/).

### 2.2. Environmental niche models

Distribution of *P. parlatorei* was modelled following maximum entropy approach using MaxEnt v. 3.4.1. A total of 76 presence records *P. parlatorei* were used as training data over the entire current distribution of the species (electronic supplementary material, table S1). Potential modern distribution of the species was modelled using 10 000 background points. Background points were randomly placed across a rectangular area extending 13–29° S and 63–74° W using MaxEnt default procedure. A minimum set of current climate (1960–1990) variables at 30 arc-sec resolution (downloaded from WorldClim 1.4, http://www.worldclim.org/current) were selected by jackknifing techniques that maximized regularized training gain (a measure of sample likelihood that describes how much better the MaxEnt distribution fits the presence data compared to a uniform distribution) and area under the ROC curve (AUC). The final model included variables Bio_18_, Bio_10_, Bio_19_, Bio_15_, Bio_4_, Bio_14_, Bio_3_ and Bio_11_ (in decreasing order of contribution; see electronic supplementary material, table S2). Regularized training gain of the final model was 3.497, training AUC 0.995.

Mid-late twenty-first century climatic conditions for 2050 (mean of 2041–2060) and 2070 (mean of 2061–2080) were derived from 13 Global Climate Models (GCMs; electronic supplementary material, table S3) for two contrasting greenhouse gas representative concentration pathways RCP2.6 and RCP8.5 (IPCC, AR5). RCP2.6 assumes that global annual greenhouse gas emissions (measured in CO_2_-equivalents) peak during 2010–2020, with emissions declining substantially thereafter. Global temperature increase averages c. 1° by 2081–2100. In RCP8.5, emissions continue to rise throughout the twenty-first century and by 2081–2100 global temperature increase by 3.7°C [43]. We used data available at WorldClim 1.4 from CMIP5 (Coupled Model Intercomparison Project Phase 5, http://www.worldclim.org/CMIP5v1) downscaled to 30 arc-sec standard bioclimatic sets [44] available in WorldClim 1.4. The final *P. parlatorei* MaxEnt model was projected onto conditions predicted by each of the GCMs to obtain 13 models of predicted suitability for the species for 2050 and 2070. Ensemble models were calculated by averaging all model outputs to obtain a consensus species suitability during 2050 and 2070 for two greenhouse gas concentration scenarios, RCP2.6 and RCP8.5.

Because BIO_18_ (precipitation of warmest quarter) and BIO_10_ (mean temperature of warmest quarter) were the bioclimatic variables with the highest contribution to the model (electronic supplementary material, table S2), we analysed for each of them the mean of 10 MaxEnt replicates univariate response curves and identified threshold conditions that defined optimum (mean probability of presence >0.4) and suboptimum (mean probability of presence <0.4). This generated range maps of dry (D), optimum (O) and wet (W) conditions for BIO_18_ and cold (C), optimum (O) and hot (H) conditions for BIO_10._ We then generated classified maps of D, O, W and C, O, H for modern future (2070) RCP2.6 and 8.5 mean conditions (based on predictions of the 13 GCMs). These layers were cross-classified within ArcGis 10.2 to obtain modern-2070 (RCP2.6 and 8.5 transition maps of conditions for both bioclimatic variables).

In order to estimate the possible fate of known *P. parlatorei* populations sampled for genetic analyses, we assigned to each of the 24 selected populations the corresponding state of BIO_18_ and BIO_10_(D, O, C and C, O, H) for the modern and 2070 periods (RCP2.6 and RCP8.5). For this, we calculated a 10 km buffer area (approx. 400 pixels) around each population coordinate and assigned the most frequent state (D, O, C and C, O, H) to each population.

## 3. Results

**Figure 1.**
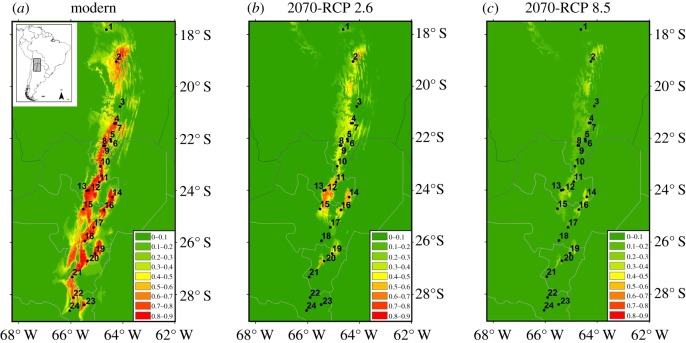
(*a*) Bioclimatic suitability (logistic output) of *Podocarpus parlatorei* in northern Argentina and southern Bolivia under modern conditions (approx. 1960–1990) based on a final model of 8 variables. (*b*) and (*c*) *P. parlatorei* mean suitability predicted by projecting model of figure 1*a* onto climatic conditions predicted by 13 global circulation models for 2070 (2061–2080) under the RCP2.6 and RCP8.5 AR5 greenhouse gas concentration trajectories, respectively. Numbered dots represent *P. parlatorei* populations listed in table 1.

Contrary to our expectations of latitudinal and/or altitudinal range shifts our results show dramatic reductions in the overall bioclimatic suitability of *P. parlatorei* across its entire range (figure 1; electronic supplementary material, figure S1). These reductions in suitability were more moderate for RCP2.6. Under this scenario, the central and northern populations retained a certain amount of suitability in 2050 and through 2070 (figure 1; electronic supplementary material, figure S1); however, the southern range of the species (population 21–24) becomes virtually unsuitable even by 2050. The scenario RCP8.5 shows a much stronger effect with overall strong reductions of suitability (*p* < 0.4) by 2070 throughout the entire species’ range (figure 1).

**Table 1.** Condition classes for BIO_10_ (temperature of the warmest quarter; C, cold; O, optimal; H, hot; figure 2) and BIO_18_ (precipitation of the warmest quarter: D, dry; O, optimal; W, wet; figure 2) during the modern period, 2070 RCP2.6, and 2070 RCP8.5 at locations (average of pixels around a 10 km radius) corresponding to *P. parlatorei* populations. Future condition ranges for RCP2.6 and RCP8.5 were calculated based on the average values of variables predicted by 13 GCMs. The population numbers correspond to locations mapped in figure 1.

#	population	BIO_10_ modern	BIO_10_ 2070 RCP2.6	BIO_10_ 2070 RCP8.5	BIO_18_ modern	BIO_18_ 2070 RCP2.6	BIO_18_ 2070 RCP8.5
1	Mizque	O	O	O	D	D	D
2	Villa Serrano	O	O	H	O	D	O
3	Monteagudo	H	H	H	D	O	O
4	Tarija 3	O	O	H	O	D	D
5	Tarija 4	H	H	H	O	O	O
6	Tarija 5	H	H	H	O	O	O
7	Tarija 7	H	H	H	O	D	O
8	El Nogalar	O	O	H	O	D	D
9	Los Toldos	O	H	H	O	D	O
10	San Andrés	O	O	O	O	D	D
11	Calilegua	O	H	H	O	D	O
12	Tiraxi	O	O	H	O	D	D
13	Tiraxi arriba	O	O	O	O	D	D
14	El Fuerte	O	H	H	O	D	O
15	San Lorenzo	O	O	H	O	D	D
16	El Rey	O	O	H	O	D	D
17	Valderrama	O	O	H	O	D	D
18	La Candelaria	O	O	H	O	D	D
19	Sierra Medina	O	H	H	O	D	O
20	Taficillo	O	H	H	O	O	O
21	La Banderita	O	H	H	O	D	D
22	Pinar Grande	O	H	H	O	D	D
23	Tintigasta	O	H	H	O	D	D
24	Concepción	H	H	H	D	D	D

**Figure 2.**
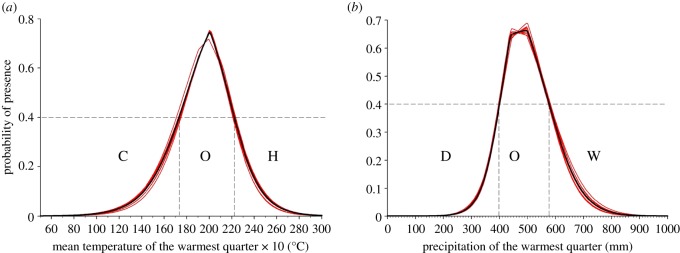
Response curves for the two variables with the highest contribution to the model (*a*) BIO_10_: temperature of the warmest quarter, and (*b*) BIO_18_: precipitation of the warmest quarter. Curves represent the predicted probability of presence (P) of *P. parlatorei* from univariate models using the corresponding variable (results of 10 replicates in red and mean in black). Dashed vertical lines represent thresholds separating optimal (O) ranges of conditions (*p* > 0.4) from non-suitable conditions (*p* < 0.4). H, hot; C, cold for BIO_10_ and W, wet; D, dry for BIO_18_.

Bioclimatic variables BIO_18_ (precipitation of the warmest quarter) and BIO_10_ (mean temperature of the warmest quarter) had the highest contribution to the model, 42.6% and 30.8%, respectively. In addition, BIO_18_ showed a very high permutation importance (74.3%) and BIO_10_ a moderate importance (17.5%; electronic supplementary material, table S2). Two relatively narrow bell-shape curves describe the probability of presence when these two variables are used individually to model the probability presence of *P. parlatorei* (figure 2)*.* Optimal (*p* > 0.4) thermal conditions occur when the mean temperature of the warmest quarter lies between 17.3°C and 22.2°C, whereas the optimal (*p* > 0.4) range of precipitation of the warmest quarter is 400–575 mm (figure 2). Hereafter we define as cold: BIO_10_ < 17.3, hot: BIO_10_ > 22.2 and thermally optimal: 17.3 ≤ BIO_10_ ≤ 22.2°C. Likewise, we define as dry: BIO_18_ < 400 mm, wet: BIO_18_ > 575 mm and optimal rainfall: 400 ≤ BIO_18_ ≤ 575 mm.

**Figure 3.**
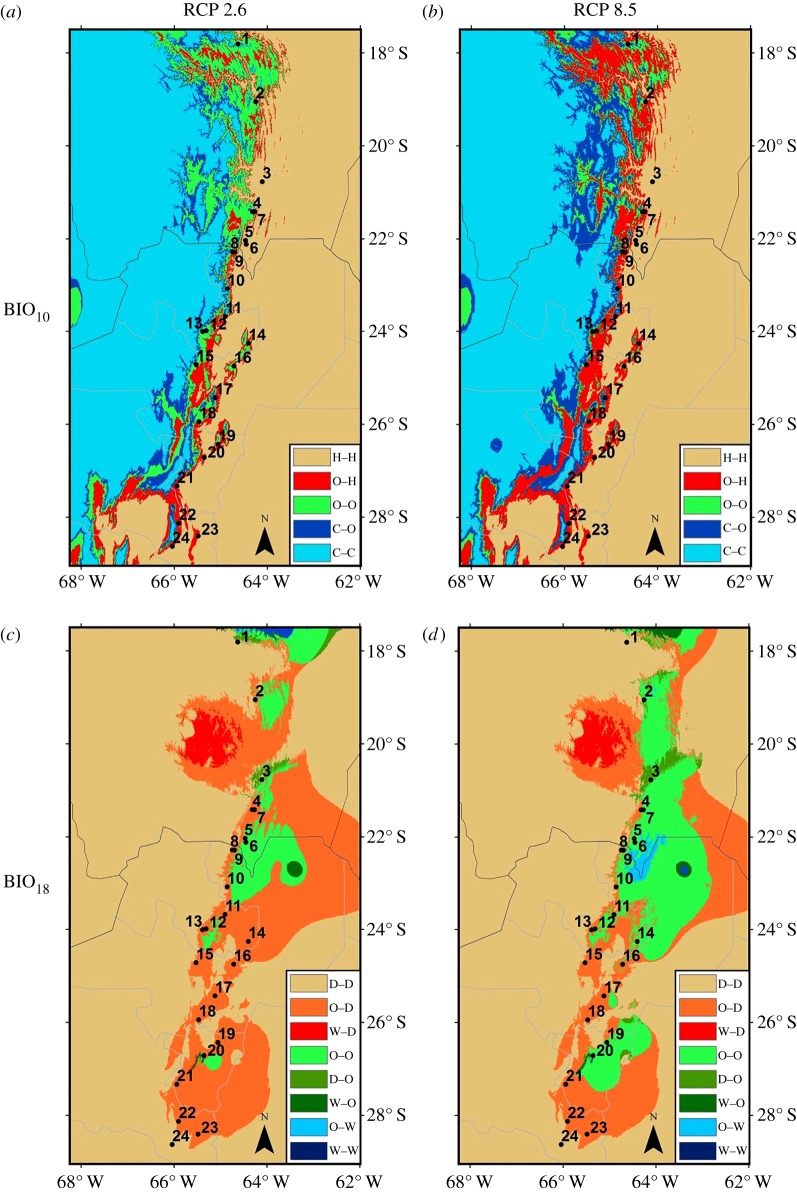
Transitions in BIO_10_ (temperature of the warmest quarter): (*a*) modern-to-2070 RCP2.6 and (*b*) modern-to-2070 RCP8.5. Condition categories: C, cold; O, optimal; H, hot (figure 2). Transitions in BIO_18_ (precipitation of the warmest quarter): (*c*) modern-to-2070 RCP2.6 and (*d*) modern-to-RCP8.5. Condition categories: D, dry; O, optimal; W, wet (figure 2). Future condition ranges for RCP2.6 and RCP8.5 were calculated based on the average values of variables predicted by 13 GCMs.

Mapping the modern-to-2070 change of these bioclimatic envelopes (figure 3) shows important spatial shifts in conditions. Thermally, high-elevation belts upwards and west of the current *P. parlatorei* distribution that are currently too cold for the species (C) will transition into thermally optimal conditions (C─O transitions, light green in figure 3*a,b*). Concomitantly, many areas at the current distribution and downslope east of the current distribution will become too warm for *P. parlatorei* (O─W transitions, red in figure 3*a,b*). Only a few areas where currently the species develops will remain thermally optimal, more so under the RCP2.6 scenario (O─O, dark green in figure 3*a*).

Changes in precipitation conditions predicted for the mid-late twenty-first century will be extensive, especially under the RCP2.6 scenario (figure 3*c*). Vast areas with currently optimal levels of warm season precipitation will transition into areas too dry to sustain *P. parlatorei* forests (O─D transitions, orange in figure 3*c,d*). Many of these areas where aridification is expected to overlap with high-elevation areas, which in turn are expected to become thermally optimal, suggests a switch in the limiting factor that induces potential range retractions rather than shifts. On the other hand, some central and northern areas that are predicted to remain rainfall-wise optimal, especially under the RCP8.5 scenario (O─O, dark green in figure 3*d*), largely overlap with areas becoming too hot for the species, again evidencing a switch in the limiting factor that prevents potential range expansions towards lowland areas.

Most extant populations of *P. parlatorei* of northwestern Argentina and southern Bolivia will towards the mid-late twenty-first century face important changes in either thermal or precipitation conditions, or both (table 1). Nineteen of the 24 populations are currently growing in optimal thermal conditions while the remaining five develop in hot environments. Of these 19, 8 (42%) are expected to become hot under the RCP2.6 scenario and 16 (84%) will do so under the RCP8.5 scenario while none will become cold. The remaining five (mostly northern) populations already in hot conditions under the current climate will remain under this condition in the future (table 1). Twenty-one of 24 populations currently develop in optimal rainfall levels while three thrive in dry conditions. Eighteen out of 21 populations (86%) currently in optimal rainfall levels will become dry under RCP2.6, whereas 12 (57%) will become dry under the RCP8.5 scenario. Of the three populations currently thriving in dry conditions, two (the northernmost and southernmost populations) will remain in the dry condition and one northerly population will reach optimal rainfall by 2070 (for both RCP2.6 and 8.5; table 1). Of all populations remaining in the future in optimal thermal condition, none will remain in future optimal rainfall conditions. Likewise, none of the populations remaining or reaching optimal rainfall levels in the future will have corresponding optimal thermal conditions (table 1).

## 4. Discussion

The models projected for 2050 and 2070 are surprising in their predictions because, contrary to expectations for latitudinal expansion, i.e. poleward migrations or altitudinal change, the subtropical *P. parlatorei* will locally shrink its range. While we cannot discard the possibility for potential upward migration as predicted for montane taxa, the evidence presented here strongly suggests that all populations of *P. parlatorei* will be affected by the new climatic conditions. It was suggested that the main risks related to projected climatic changes for the twenty-first century are linked to either high variation of local climates, development of novel climates, or the disappearance of extant climates [45]. Our results are in concert with the significant modification as well as the loss for local conditions suitable for *P. parlatorei* persistence under changing climates. Therefore, the local extinction of *P. parlatorei* populations seems to be the inevitable outcome under the tested scenarios. We also hereby show that the combined effects of changing temperatures and the modification of precipitation regimes may lead to unexpected and previously underestimated local ecological responses.

Modelling and global studies predicted that changes in climate may be a significant extinction driver in the tropics [46] and particularly for the tropical Andes high plant extinction rates are expected [47]. Tropical species may be especially susceptible to 21st century changing climates due to the fact that communities have evolved under less variable temperatures at distinct temporal scales than those inhabiting high-latitude climates. This high sensitivity to temperature has been already measured in the field showing decelerating growth of tropical trees with subtle increases in average temperature [48] and also reduced precipitation [49]. Species with an overall wide distribution but locally restricted to mountain habitats of the northern Andes may suffer increase risk of extinction. This is the case of *P. parlatorei*, the only conifer of Yungas forests with a key role in the community and ecosystem, which adds urgency to the design of conservation actions.

The future model predicts that southern populations of the distribution will have a combination of precipitation and temperature that will cause the species to become locally extinct due to hydric and thermal stress intolerance. While in the northern part of the distribution, although one of the most relevant variables of the model, i.e. summer temperature and precipitation, may go into an unfavourable state and the other may not, the condition of both favourable (optimal) variables for the species would not exist. Thus, only the combination of hot temperature and optimum precipitation or optimum temperature and dry climate can be achieved. These combinations of increased precipitation and temperature may facilitate the entry of neotropical species (tolerant to hot climates) that would outcompete *P. parlatorei*.

Specifically, we can affirm a general trend of squeeze of the entire *P. parlatorei* range, because the conditions will vary locally, so that precipitation and temperature have an additional effect that can be exacerbated at particular local areas, to the detriment of the species' environmental niche. Effectively, *P. parlatorei* is affected by precipitation and the temperature of the warmest quartile, its optimum condition being the combination of moderate precipitation (400–575 mm) and mean temperature (17.5°–22.2°C) (figure 2). Throughout *P. parlatorei* distribution the temperature condition is determined by elevation, while the wet season is associated with wet summers. However, if the summer becomes dry or warmer, the environmental niche is lost. As the species inhabits a narrow mountainous area, and is already at the treeline, *P. parlatorei* would only survive at microsites, which will represent refuges for cold-tolerant species inhabiting low latitudes.

In previous analysis [38,41,50], we described genetic patterns along the *P. parlatorei* range. Northern populations show reduced genetic diversity, which may negatively affect adaptability, increasing stress intolerance and susceptibility to diseases, thus limiting their capacity to respond to future scenarios. Severe reductions in suitability are also predicted for central populations and consequently the concomitant genetic losses, given that some of these populations hold unique isozyme variants (e.g. 15. San Lorenzo population) or exclusive chloroplast DNA haplotypes (e.g. 10. San Andres population) [50]. Hence, possible local extinction of these populations prompts an aggravating factor that may potentially impact on the species’ gene pool due to the loss of unique genetic variants and elevated haplotype diversity present in this area [50]. A critical situation occurs towards the southern distribution because the model predicts total loss of optimal conditions (table 1). Populations located in an area between 22 and 23° S latitude are genetically heterogeneous and hold relatively high genetic diversity [38,50] and one unique chloroplast haplotype [41]. Thus future *de novo* establishment will be limited and the forest will probably be maintained by surviving adult trees. Under this new scenario, and considering that *P. parlatorei* is a cold-tolerant species (or at least sensitive to warm), high-elevation southernmost areas should be considered the first priority in conservation actions as they could function as refuge areas for this and other species ecologically associated with this currently cold subtropical forest.

*Podocarpus parlatorei*, under future scenarios, is between ‘the sword and the wall’ because the western sector is compressed by the entrance of the Chaco biome, tolerant to high temperatures and low humidity, while in the eastern sector it is closer to the desert of the Puna and Prepuna, where the temperature is low. This determines a future scenario that will harbour populations withstanding suboptimal conditions because little chance exists for the required climatic combinations that make up the environmental niche of *P. parlatorei*.

### 4.1. Climatic considerations

Contemporary plant range changes are most frequently reported for mountain regions, with upward elevational shifts of the mountain treeline being the most commonly documented response to increasing temperatures [51,52]. In an experimental study, Feeley *et al*. [53] detected upslope shifts in response to temperature changes in 38 Andean tree genera. Also future ecological niche modelling for *Alnus acuminata* predicts losses in the low-elevation areas of the montane cloud forest and gains at higher altitudes in response to higher temperature. An upward shift of premontane forest was suggested as a general response of all Yungas vegetation strips, so that a general increase in the altitudinal levels was expected along the Andes [54,55]. Evidence of habitat reduction due to climate change in Andean environments has also been reported for bird species [46], and anomalies have been identified in the increase in trunk growth diameters due to changes in CO_2_ concentration [48]. From the many climate change studies, it was shown that predicted responses of taxa will depend on species' tolerance to rising temperatures and their ability to migrate, combined with anthropogenic land degradation and/or reduced availability of suitable habitats, resulting in rapid losses of potential areas and high risks of extinction [16,20,46,52,53,56], at least locally. The aggravated situation of southern *P. parlatorei* populations is even more complex, because they have no places to move as they are already occupying the top of the mountains.

In general, in most climate change studies, the displacement of species is attributed to changes in temperature, and for taxa inhabiting altitudinal gradients the upward displacement hypothesis prevails in response to thermal conditions [57]. The effect of changes in precipitation has been apparently less considered, as it results from complex effects [57]. However, in our focal species, the precipitation of the warmest quartile has the highest percentage contribution to the model, and this combined with the temperature of the warmest quartile are the variables that most strongly (i.e. 73% contribution to the model) condition suitability. Therefore, in a mountain environment of subtropical latitudes, whether the species is characterized as cold tolerant or not apparently does not imply that thermal changes are the ones that most strongly modify its environmental niche, but rather water stress during hot periods.

### 4.2. Conservation actions

Populations are the relevant units for evolutionary processes and ecological functioning [58,59]. The interpretation of ENNMs results in light of genetic data can contribute to the identification of wild populations of *P. parlatorei*, which may be considered conservation priorities under a climate change scenario, as a northern Monteagudo and southern Concepcion population that harbour unique haplotypes and in turn are already under unsuitable hot and dry conditions. The most drastic situation is predicted for southern populations that will be susceptible to local extinction by low precipitation and high temperatures. The southern area is considered a hotspot of endemicity and its inclusion in a system of protected areas has been previously recommended [60,61]. We suggest prioritizing conservation actions in both current and future areas of importance for *P. parlatorei,* as suggested by Altamirano *et al*. [62]. Along with the loss of southern subtropical *P. parlatorei*, the whole mountain ecosystem will be threatened, because this conifer characterizes montane forests and many species depend on it such as epiphytes, ferns and birds [36]. Thus, cloud forests will probably remain as relict vegetation in more humid physiographical units, such as ravines on southern slopes. The collapse of an entire community can exceptionally occur with the demise of a single ‘keystone’ species, such as *P. parlatorei*, which is the dominant unique conifer of tropical-subtropical montane habitats. Loss of native forest is a key conservation concern globally, for reasons of biodiversity conservation, climate change and ecosystem services [63]. Distribution models, developed with calibrated climate variables [64], agree with the general pattern of future retraction and upward migration of premontane forest along the Andes. Thus, long-term germplasm preservation of *P. parlatorei* populations is recommended by maintaining a seed bank or through *ex situ* cultivation of specimens in arboreta as well as urgent *in situ* conservation actions [65], particularly of populations outside protected areas, in order to counteract inbreeding and maladaptive effects [60].

In relation to other *Podocarpus* species that have been analysed under future change scenarios and genetic characteristics, the work of Mellick *et al*. [66] on *Podocarpus elatus* (east coast of Australia) also shows evidence of a change in range, where northern populations would move further north and south, and southern populations would be reduced in size and confined to a small portion of the south coast. Therefore, *Podocarpus* species are susceptible to climate change and at least in the past, have been able to trace their optimum. It will be a challenge for the future to keep pace with climate change.

### 4.3. Conclusion

A high consensus among 13 future climate models shows that *P. parlatorei* will experience an overall range squeeze of its currently occupied total area due to combined drying/warming trends that prevent the opening of new suitable areas for the species, thus substantially threatening the persistence of the species as a whole. Given that loss of native forest is a key conservation concern globally, urgent measures are needed to conserve *P. parlatorei* in order to preserve the ecosystem services that such a unique conifer provides in montane forests along the subtropical Andes.

## Supplementary Material

Supplementary material Quiroga et al.

## Supplementary Material

Figure S1

## Data accessibility

Electronic supplementary material, tables S1, S2 and S3 and figure S1.
