# Supplementary material for: Niche squeeze induced by climate change of the cold-tolerant subtropical montane Podocarpus parlatorei
Source: R Soc Open Sci. 2018 Nov 28;5(11):180513. doi: 10.1098/rsos.180513 (PMC6281919; doi:10.1098/rsos.180513)
Supplement: Supplementary material Quiroga et al. [file rsos180513supp1.docx]

**Supplementary material Quiroga et al.** Niche squeeze induced by climate change of the cold-tolerant subtropical montane *Podocarpus parlatorei*

**Figure S1**: (**A**) Bioclimatic suitability (logistic output) of *Podocarpus parlatorei* in northern Argentina and southern Bolivia under modern conditions (~1960-1990) based on a final model of 8 variables. (**B**) and (**C**) *P. parlatorei* mean suitability predicted by projecting model of Fig 1A onto climatic conditions predicted by 13 global circulation models for 2050 (2041-2060) under the RCP2.6 and RCP8.5 AR5 greenhouse gas concentration trajectories, respectively. Numbered dots represent *P.parlatorei* populations listed in Table 1.

**Table S1**. *P. parlatorei* presence records used as training points for the MaxEnt model.

| W Longitude | S Latitude |  | W Longitude | S Latitude |
| --- | --- | --- | --- | --- |
| -66.040100 | -28.626690 |  | -64.483330 | -21.683330 |
| -65.482890 | -28.402400 |  | -64.100000 | -21.666670 |
| -65.901210 | -28.131140 |  | -64.833330 | -21.533330 |
| -65.938460 | -27.332890 |  | -64.316670 | -21.416670 |
| -65.916670 | -27.216670 |  | -64.316670 | -21.416670 |
| -65.353550 | -26.718460 |  | -64.266600 | -21.416670 |
| -65.053760 | -26.431570 |  | -64.316666 | -21.416666 |
| -65.468911 | -25.945428 |  | -64.533330 | -21.016670 |
| -65.116700 | -25.430000 |  | -64.366670 | -20.833330 |
| -64.713900 | -24.750500 |  | -64.033330 | -20.283330 |
| -65.525020 | -24.715140 |  | -64.300000 | -20.200000 |
| -65.533330 | -24.500000 |  | -64.316670 | -19.566670 |
| -64.470760 | -24.296400 |  | -64.250000 | -19.050000 |
| -64.401900 | -24.258470 |  | -64.316670 | -19.000000 |
| -65.383330 | -24.016670 |  | -64.166670 | -18.900000 |
| -65.386420 | -24.010420 |  | -63.883330 | -18.816670 |
| -65.308100 | -23.990200 |  | -63.900000 | -18.733330 |
| -65.308060 | -23.990180 |  | -63.250000 | -18.683330 |
| -64.901060 | -23.681620 |  | -63.016670 | -18.666670 |
| -64.900000 | -23.666670 |  | -64.033330 | -18.650000 |
| -64.940680 | -23.651840 |  | -64.028890 | -18.609170 |
| -64.900000 | -23.650000 |  | -63.033330 | -18.600000 |
| -64.916670 | -23.616670 |  | -63.100000 | -18.583330 |
| -64.866670 | -23.100000 |  | -64.100000 | -18.566670 |
| -64.855700 | -23.076100 |  | -64.083330 | -18.550000 |
| -64.750000 | -22.466670 |  | -63.950000 | -18.533330 |
| -64.716670 | -22.350000 |  | -64.106110 | -18.483330 |
| -64.697600 | -22.283330 |  | -64.500000 | -18.450000 |
| -64.745500 | -22.279400 |  | -65.383330 | -18.266670 |
| -64.745550 | -22.279370 |  | -65.366670 | -18.233330 |
| -64.450000 | -22.116670 |  | -63.956110 | -18.100000 |
| -64.466666 | -22.033333 |  | -65.483330 | -17.850000 |
| -64.650000 | -22.016670 |  | -65.476100 | -17.815300 |
| -64.683330 | -21.900000 |  | -65.406110 | -17.766670 |
| -64.316670 | -21.900000 |  | -65.366670 | -17.716670 |
| -64.850000 | -21.716670 |  | -65.722780 | -17.426670 |
| -64.483330 | -21.700000 |  | -66.633330 | -16.766670 |
| -64.483330 | -21.683330 |  | -68.083330 | -16.516670 |
| -64.100000 | -21.666670 |  | -65.053764 | 26.431574 |
| -64.833330 | -21.533330 |  |  |  |
| -64.316670 | -21.416670 |  |  |  |

**Table S2**. Estimates of relative contributions of the environmental variables to the MaxEnt model based on monitoring regularized training gain changes upon addition or random permutation of each variable.

| **Variable** | **Percent contribution** | **Permutation importance** |
| --- | --- | --- |
| BIO_18_ | 42.6 | 74.3 |
| BIO_10_ | 30.8 | 17.5 |
| BIO_19_ | 11.5 | 0 |
| BIO_15_ | 8.2 | 2 |
| BIO_4_ | 6.5 | 2.4 |
| BIO_14_ | 0.3 | 2.6 |
| BIO_3_ | 0.1 | 0.3 |
| BIO_11_ | 0 | 1 |

**Table S3**. List of General Circulation Models used for estimating mid-21^st^ century climatic conditions and future *P. parlatorei* suitability.

| **GCM Model** | **Code** |
| --- | --- |
| BCC-CSM1-1 | BC |
| CCSM4 | CC |
| CNRM-CM5 | CN |
| GFDL-CM3 | GF |
| GISS-E2-R | GS |
| HadGEM2-AO | HD |
| HadGEM2-ES | HE |
| IPSL-CM5A-LR | IP |
| MRI-CGCM3 | MG |
| MIROC-ESM-CHEM | MI |
| MPI-ESM-LR | MP |
| MIROC-ESM | MR |
| NorESM1-M | NO |
