# Supplementary figures and images for: Niche squeeze induced by climate change of the cold-tolerant subtropical montane Podocarpus parlatorei
Source: R Soc Open Sci. 2018 Nov 28;5(11):180513. doi: 10.1098/rsos.180513 (PMC6281919; doi:10.1098/rsos.180513)

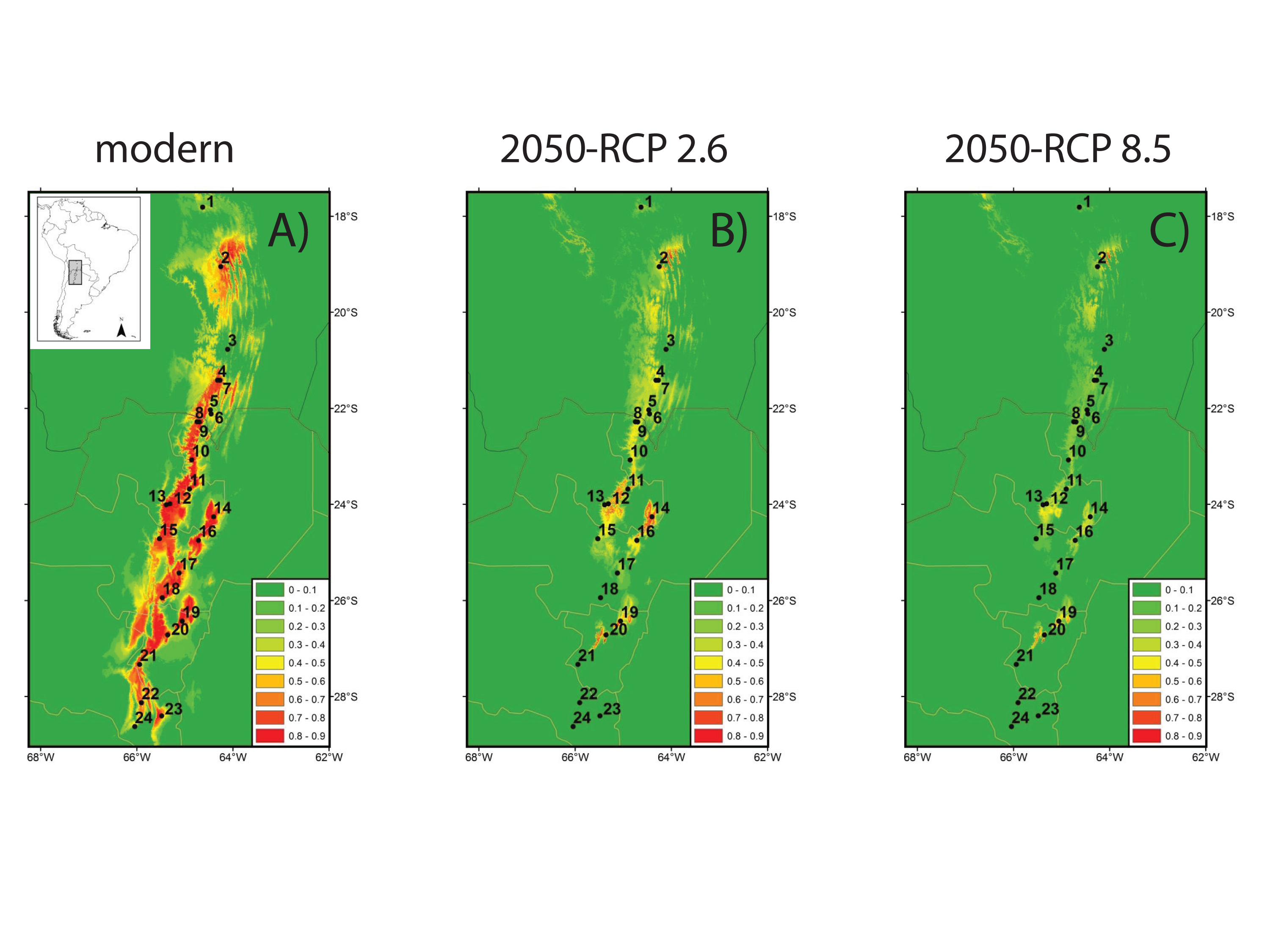

Supplement: Figure S1 [file rsos180513supp2.tif]
